# Uncovering the Hidden World of Aqueous Humor Proteins for Discovery of Biomarkers for Marfan Syndrome

**DOI:** 10.1002/advs.202303161

**Published:** 2023-12-13

**Authors:** Yumeng Shi, Jiahui Chen, Lei Cai, Xueling Zhang, Zexu Chen, Jin Yang, Yongxiang Jiang, Yi Lu

**Affiliations:** ^1^ Eye Institute and Department of Ophthalmology, Eye and ENT Hospital Fudan University Shanghai 200031 China; ^2^ NHC Key Laboratory of Myopia Fudan University Shanghai 200031 China; ^3^ Key Laboratory of Myopia Chinese Academy of Medical Sciences Shanghai 200031 China; ^4^ Shanghai Key Laboratory of Visual Impairment and Restoration Shanghai 200031 China

**Keywords:** aqueous humor proteomics, DIA, ectopia lentis, FAIMS, Marfan syndrome

## Abstract

Ectopia lentis is a hallmark of Marfan syndrome (MFS), a genetic connective tissue disorder affecting 1/5000 to 1/10 000 individuals worldwide. Early detection in ophthalmology clinics and timely intervention of cardiovascular complications can be lifesaving. In this study, a modified proteomics workflow with liquid chromatography‐tandem mass spectrometry (LC‐MS/MS)‐based data‐independent acquisition (DIA) and field asymmetric ion mobility spectrometry (FAIMS) to profile the proteomes of aqueous humor (AH) and lens tissue from MFS children with ectopia lentis is utilized. Over 2300 and 2938 comparable proteins are identified in AH and the lens capsule, respectively. Functional enrichment analyses uncovered dysregulation of complement and coagulation‐related pathways, collagen binding, and cell adhesion in MFS. Through weighted correlation network analysis (WGCNA) and machine learning, distinct modules associated with clinical traits are constructed and a unique biomarker panel (Q14376, Q99972, P02760, Q07507; gene names: GALE, MYOC, AMBP, DPT) is defined. These biomarkers are further validated using advanced parallel reaction monitoring (PRM) in an independent patient cohort. The results provide novel insights into the proteome characterization of ectopia lentis and offer a promising approach for developing a valuable biomarker panel to aid in the early diagnosis of Marfan syndrome via AH proteome.

## Graphical abstract

The rare disease Marfan syndrome (MFS) is explored using advanced mass spectrometry. Over 2000 aqueous humor proteins in pediatric patients are discovered, with an investigation into clinical correlations and a potential biomarker panel for early MFS detection. This study reveals valuable insights into MFS pathophysiology, enriching our understanding of the intricate ocular and systemic interplay.

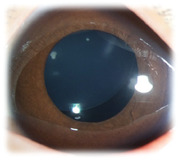

## 1 Introduction

Marfan syndrome (MFS) is an autosomal dominant connective tissue disease with a prevalence of 1/5000 to 1/10 000 and is characterized by extensive disorders of mesodermal tissues, including abnormalities of the ocular, skeletal, and cardiovascular systems.^[^ ^1^, ^2^ ^]^ Notably, it is also an age‐related disease, meaning that the complications gradually progress with age.^[^ ^3^ ^]^ The cardiovascular system, in particular, is responsible for the fact that the average natural life expectancy of MFS patients was only 32 years when there was no effective treatment.^[^ ^4^, ^5^ ^]^ The thoracic aortic disorder generally starts as an asymptomatic enlargement of the aortic root and progresses to an aneurysm over time. As the aneurysm grows, it becomes unstable and may eventually result in an acute ascending aortic dissection, a potentially fatal consequence that can shorten lifespan.^[^ ^3^ ^]^ Thus, the gradual and asymptomatic nature of such lethal complications renders diagnosis at an early stage challenging. Therefore, there is a pressing need for maximally multidimensional diagnostic strategies to screen patients in at‐risk populations as early as possible. Importantly, ectopia lentis (that is, luxation or subluxation of the lens) is a stable and characteristic manifestation of MFS. Approximately 60% of patients present with ectopia lentis at the age of 3 to 5 years.^[^ ^6^, ^7^, ^8^, ^9^ ^]^ Most children initially present to ophthalmologists' clinics with poor or blurred vision. This finding has prompted our group to focus on those pediatric cases, with the goal of identifying potential patients, and cardiovascular follow‐up will then be advised to parents so that timely therapeutic intervention may save lives.

Aqueous humor (AH), a transparent fluid nourishing the avascular tissues in the anterior segment of the eyes, is essential for many physiological functions.^[^ ^10^, ^11^ ^]^ AH sampling and analysis have been frequently used in clinical practice to aid the diagnosis and treatment of ocular diseases.^[^ ^12^ ^]^ AH proteins are derived from not only the lens and ciliary body but also filtered plasma across the blood‐ocular barrier.^[^ ^13^ ^]^

High‐accuracy mass spectrometry (MS)‐based quantitative proteomics is a promising approach for discovering new biomarkers since it is particularly effective at recognizing changes in protein abundance levels in various specimens.^[^ ^14^, ^15^ ^]^ In the context of complicated diseases, MS‐based proteomics could move the emphasis from individual proteins to biomarker panels. However, proteomic analysis of AH has proven difficult due to the extremely limited volume collected, low protein concentrations, and wide dynamic range of protein levels, resulting in less in‐depth coverage and lower throughput compared to other body fluids, such as the cerebrospinal fluid, urine, or plasma.^[^ ^16^, ^17^ ^]^

Recent technical advances in data‐independent acquisition (DIA) mode in conjunction with field asymmetric ion mobility spectrometry (FAIMS) interface have significantly improved the detectability of low‐abundance proteins.^[^ ^18^ ^]^ Our team has recently integrated these state‐of‐the‐art technologies into a modified workflow, extended the pipeline of biomarker discovery, and employed high‐throughput MS‐based proteomics to acquire proteome profiles of children's AH and lens tissue.

Here, we improved the depth of protein coverage by a large amount and first provided proteome profiling of the children with ectopia lentis. More specifically, we detected over 2300 proteins from minimal sample amounts both in AH and the anterior lens capsule. By employing weighted correlation network analysis (WGCNA) and machine learning, we characterized the proteome of the two biofluids, defined unique protein panels, and constructed the module associated with MFS clinical traits. Taken together, our findings provide evidence that modern quantitative MS‐based proteomics can serve as a clinically useful approach for identifying potential biomarkers of MFS as well as provide insights into molecular processes related to MFS and/or FBN1 mutations.

## 2 Experimental Section

### 2.1 Clinical Cohorts and Ophthalmologic Examinations

The current study was a case‐control investigation that included 32 cases and 31 controls, encompassing both the discovery and validation cohorts. The cases were MFS patients with confirmed FBN1 gene mutation recruited from a long‐term case series study on ectopia lentis conducted by Eye and Ear, Nose and Throat Hospital of Fudan University, Shanghai, from 2017 to 2022. The controls were age‐matched cataract patients without ectopia lentis or any other ocular comorbidities. All participants’ guardians were given a verbal description of the study before enrollment and completed an informed consent form. The study procedures adhered to the tenets of the Helsinki Declaration.

After obtaining a detailed history, each participant underwent systemic ophthalmological examinations including refraction, slit‐lamp, and fundus examination. The anterior segmental parameters including axial length (AL), flat keratometry value (K1), steep keratometry value (K2), mean keratometry value (Km), cylinder diopter (Cyl), axis, and anterior chamber depth (ACD) were assessed with a biometer (IOL Master 700, Carl Zeiss Meditec, Jena, Germany) at a sitting position. All patients underwent phacoemulsification and intraocular lens implantation. AH samples (20–100 µL) and the anterior lens capsule were collected during the surgery.

### 2.2 Sample Preparation

#### 2.2.1 Protein Extraction

The lens capsules were sonicated on ice using a high‐intensity ultrasonic processor (Scientz, Ningbo, China) in lysis buffer (8 M urea, Sigma‐Aldrich; 1% protease inhibitor cocktail, Merck Millipore). The cell debris was removed by centrifugation at 12 000 g at 4 °C for 10 min and the supernatant was collected. Protein concentration was determined with a BCA kit according to the manufacturer's instructions (Beyotime, Shanghai, China).

#### 2.2.2 Trypsin Digestion

For digestion, the protein solution was reduced with 5 mM dithiothreitol (Sigma‐Aldrich) for 30 min at 56 °C and alkylated with 11 mM iodoacetamide (Sigma‐Aldrich) for 15 min at room temperature in darkness. The protein sample was then diluted by adding 100 mM Tetraethylammonium bromide (TEAB, Sigma‐Aldrich) to urea concentration less than 2 M. Finally, trypsin (Promega) was added at 1:50 trypsin‐to‐protein mass ratio for the first digestion overnight and 1:100 trypsin‐to‐protein mass ratio for a second 4 h digestion. Finally, the peptides were desalted by the C18 SPE column.

#### 2.2.3 High‐Performance Liquid Chromatography (HPLC) Fractionation (For AH Samples Only)

The sample was fractionated by high pH reverse‐phase HPLC using Agilent 300 Extend C18 column (5 µm particles, 4.6 mm ID, 250 mm length). The analysis was conducted at a wavelength of 214 nm with a column temperature of 35 °C. Prior to sample injection, the chromatographic column was equilibrated with 95% buffer A (a water solution containing 5% acetonitrile, ThermoFisher Scientific) for 30 minutes until the baseline stabilized. Subsequently, a gradient elution method was initiated, and peptide samples were introduced to the HPLC system. Sample separation was carried out using a 1‐minute per tube collection interval. Fractions 11 to 46, totaling 36 fractions, were collected and subsequently combined into 12 fractions, followed by vacuum drying.

#### 2.2.4 Liquid Chromatography‐Tandem Mass Spectrometry (LC‐MS/MS) Analysis

The tryptic peptides were dissolved in mobile phase A (containing 0.1% formic acid, Fluka, and 2% acetonitrile, ThermoFisher Scientific) and separated on Vanquish neo (for AH samples) or EASY‐nLC 1200 (for lens capsule samples) ultra‐performance liquid chromatography (UPLC). Mobile phase B consisted of a solution with 0.1% formic acid and 90% acetonitrile. The liquid gradient was programmed as follows: 0–16 min, 7%−20% B; 16–24 min, 20%−32% B; 24–27 min, 32%−80% B; 27–30 min, 80% B, with a constant flow rate of 500 nl/min (for AH samples). 0–68 min, 6%∼23% B; 68–82 min, 23%∼32% B; 82–86 min, 32%∼80% B; 86–90 min, 80% B, with a constant flow rate of 500 nl/min (for lens capsule samples). The peptides were subjected to a nano‐spray ionization source followed by tandem mass spectrometry (MS/MS) in Orbitrap Exploris 480 Mass Spectrometer equipped with a high FAIMS Pro interface (ThermoFisher Scientific, Bremen, Germany).

MS spectra of lens capsule samples were acquired with a data‐dependent acquisition (DDA) mode. The top 25 precursors were sequentially isolated and fragmented in higher‐energy collisional dissociation (HCD) with 27% collision energy. FAIMS compensation voltage (CV) was set to −45 V, and −65 V. Automatic gain control (AGC) was set at 100%, with an intensity threshold of 5E4 ions/s and a maximum injection time of “Auto.” The raw MS data were processed using Thermo Proteome Discoverer (v2.4.1.15). The database utilized was “Homo_sapiens_9606_SP_20220107.fasta,” comprising 20376 protein sequences. To assess and control for false positive identifications resulting from random matches, a decoy database was included. Additionally, a common contaminant database was incorporated into the search to mitigate the impact of contaminant proteins on identification results. The enzyme cleavage specificity was set to Trypsin (Full), allowing for up to 2 missed cleavage sites. The minimum peptide length was defined as 6 amino acid residues, with a maximum of 3 variable modifications allowed per peptide. The tolerance for mass errors was set at 10 ppm for the precursor ions and 0.02 Da for the fragment ions. Fixed modification Carbamidomethyl (C) was applied, while variable modifications included Oxidation (M), Acetyl (N‐terminus), Met‐loss (M), and Met‐loss+acetyl (M). False Discovery Rates (FDR) for protein, peptide, and Peptide‐Spectrum Match (PSM) identifications were all established at 1%.

MS spectra of AH samples were acquired with DIA mode, where HCD collision energy was set to 25,30,35. FAIMS CV was set to −45 V, −70 V. AGC was set at 3E6 ions/s with a maximum injection time of “Auto.” The resulting MS data were processed using Spectronaut (V16.3) with default software parameters. The database utilized for this analysis was “Homo_sapiens_9606_SP_20 230 103,” containing 20389 protein sequences. Trypsin/P was selected as the enzyme cleavage specificity with up to 2 missed cleavage sites allowed. C modification was designated as a fixed modification for cysteine residues, while variable modifications included oxidation of methionine residues and acetylation at the N‐terminus of proteins. A decoy database was introduced to calculate the FDR resulting from random matches. FDR thresholds of 1% were applied for protein, peptide, and PSM identifications.

#### 2.2.5 AH Spectral Library Generation

To maximize the protein coverage identified in pooled samples and enhance the depth of identification in formal samples, peptides were uniformly extracted from 53 AH samples. An equal amount of peptides was obtained from each sample, totaling 3.8 µg per sample. These peptides were used to create a pooled sample with a total quantity of 200 µg for library construction. The previously described HPLC gradient method was employed for peptide separation. Peptides, dissolved in mobile phase A, were separated using the EASY‐nLC 1200 UPLC. The liquid gradient was programmed as follows: 0–16 min, 7%−20% B; 16–24 min, 20%−32% B; 24–27 min, 32%−80% B; 27–30 min, 80% B, with a constant flow rate of 500 nl/min. Following separation by the UPLC system, peptides were ionized in the NSI source and then analyzed in Orbitrap Exploris 480 Mass Spectrometer equipped with a high FAIMS Pro interface (ThermoFisher Scientific, Bremen, Germany). The ion source voltage was set to 2300 V, and FAIMS CV were ‐45 V and ‐70 V. Both precursor ions and their secondary fragments were detected and analyzed in the high‐resolution Orbitrap. The first mass spectrometry scan ranged from 400–1200 m/z with a scan resolution of 60000. The secondary mass spectrometry scan had a fixed starting point at 110 m/z with a resolution of 30000, and TurboTMT was turned off. Data acquisition was performed using the DDA approach. This involved selecting the top 15 precursor ions with the highest signal intensity following the first scan, subjecting them sequentially to HCD with 27% collision energy, and subsequently conducting secondary mass spectrometry analysis. To optimize mass spectrometry efficiency, AGC was set at 75%, the signal threshold was 10000 ions/s, and the maximum injection time was 100 ms. Dynamic exclusion for tandem mass spectrometry scans was set at 30 s to prevent repeated scanning of precursor ions and enhance spectral utilization.

For DDA data analysis, the embedded Pulsar search engine within Spectronaut (v 16.3) was utilized with default software parameters. The database employed for this analysis consisted of 20389 protein sequences from “Homo_sapiens_9606_SP_20 230 103.” A decoy database was included to assess the FDR resulting from random matches. The enzyme cleavage specificity was set to Trypsin/P, allowing for up to 2 missed cleavage sites. The minimum peptide length was defined as 7 amino acid residues and a maximum of 5 variable modifications were permitted per peptide. Carbamidomethyl (C) was designated as a fixed modification for cysteine residues, while variable modifications included the oxidation of methionine residues and acetylation at the N‐terminus of proteins. The FDR for protein, peptide, and PSM identifications was established at 1%.

#### 2.2.6 Quantitative Analysis and Differential Protein Selection

For DIA proteomic quantitative analysis (AH), search results yield the Normalized Intensity for each protein across diverse samples (the protein's original intensity values normalized across samples). Relative Quantification (R) for proteins across various samples is derived through a centering transformation on the Normalized Intensity (I). The calculation formula is as follows, where i represents the sample, and j represents the protein:

(1) $$R_{ij}=I_{ij}/\mathrm{Mean}\left(I_{j}\right)$$

Similarly, in Label‐Free proteomic quantitative analysis (lens capsule), search results provide the LFQ (Label‐Free Quantification) Intensity for each protein across different samples (the protein's original intensity values normalized across samples). Relative Quantification (R) for proteins across diverse samples is acquired by centering the LFQ Intensity (I). The calculation formula is as follows, where i represents the sample, and j represents the protein:

(2) $$R_{ij}=I_{ij}/\mathrm{Mean}\left(I_{j}\right)$$

To assess differential protein expression between sample groups, the Fold Change (FC) is calculated as the ratio of the mean relative quantitative values for each protein across multiple replicate samples. For example, when comparing sample group A to sample group B, the formula is as follows, where R represents the relative quantitative values of proteins, i refers to samples, and k pertains to proteins:

(3) $$FC_{A/B,k}=\mathrm{Mean}\left(R_{ik},i\in A\right)/\mathrm{Mean}\left(R_{ik},i\in B\right)$$

The statistical significance of these differences is determined by conducting a t‐test on the relative quantitative values of each protein within the compared sample groups. The resulting P‐value, with a default threshold of P‐value < 0.05, serves as the measure of significance. To meet the normal distribution assumptions required for the t‐test, the relative quantitative values of proteins undergo a Log2 transformation prior to testing, following this formula:

(4) $$P_{k}=T\cdot \mathrm{test}\left(\log2\left(R_{ik},i\in A\right),\;\log2\left(R_{ik},i\in B\right)\right)$$

In this differential analysis, protein expression changes are deemed significant when the P‐value is less than 0.05. Changes exceeding a 1.5‐fold increase are considered significant upregulation, while changes less than 1/1.5‐fold are considered significant downregulation.

#### 2.2.7 Protein Annotation and Functional Enrichment

We annotated the subcellular structure of the protein using WoLF PSORT software (http://www.genscript.com/psort/wolf_psort.html) The Pfam database (http://pfam.xfam.org/) was used for protein domain enrichment analysis. Gene Ontology (GO) and Kyoto Encyclopedia of Genes and Genomes (KEGG) databases were used for GO categories and KEGG pathway enrichment analysis. Fisher's exact test was used to analyze the significance of the above functional enrichment of differentially expressed proteins (DEPs) (using the identified protein as the background). A *p*‐Value < 0.05 was considered significant. For further hierarchical clustering based on differentially expressed protein functional classification, we first collated all the categories obtained after enrichment along with their *p*‐Values and then filtered for those categories that were at least enriched in one of the clusters with a *p*‐Value < 0.05. This filtered *p*‐Value matrix was transformed by the function *x* = −log10 (*p*‐Value). These *p*‐Values were then clustered by one‐way hierarchical clustering (Euclidean distance, average linkage clustering) in Genesis. Cluster membership was visualized by a heat map using the “heatmap” function from the “ggplot2” R‐package.

#### 2.2.8 Protein‐phenotype Correlation Analysis

We performed WGCNA to cluster proteins with similar expression patterns, that is, modules, and investigate their association with specific ocular traits. The co‐expression network construction process involved the following steps: First, hierarchical clustering analysis was conducted based on protein expression to detect outliers. Next, a weighting coefficient, β, was selected to establish the adjacency matrix and achieve a network with scale‐free topology characteristics. Then, modules were defined as branches of a cluster tree using hierarchical clustering and dynamic tree‐cutting methods. The R package WGCNA was used to construct the network (version 1.69).

To identify key proteins, we employed a two‐step approach. Firstly, we calculated the Pearson correlation coefficients between the module eigengenes (MEs) and clinical traits to identify modules significantly associated with the clinical trait (P < 0.05). Secondly, we calculated the Pearson correlation coefficients between the protein expression levels and clinical traits [Gene significance (GS)]. We defined the top 10 hub proteins with the highest absolute value of GS in each clinically significant module. Hub proteins were then searched against the STRING database version 11.5 for protein‐protein interactions (PPIs). Functional enrichment analyses were conducted by the corresponding database as described above.

#### 2.2.9 Machine Learning Strategy

In the field of machine learning, feature selection plays a crucial role in improving model performance and preventing overfitting. In this study, we employed a feature screening process to eliminate irrelevant and redundant features, thus reducing the risk of unstable model outcomes and poor generalization ability. Firstly, we excluded features with a missing rate greater than 50% and imputed the missing values of the remaining data using the k‐nearest neighbor (KNN) algorithm.^[^ ^19^, ^20^ ^]^ Next, we removed features with zero variance, as they provided no predictive value. To mitigate the effect of collinearity between features, we computed the Pearson correlation coefficient between features, and highly correlated features (r > 0.8) were filtered such that only the feature with the highest correlation with sample classification was retained. The resultant set of 770 features was used for subsequent machine‐learning analyses.

To construct a classifier, we utilized the support vector machine (SVM) algorithm and divided the dataset into training and test sets using hierarchical sampling.^[^ ^20^ ^]^ Model performance evaluation metrics, including sensitivity, specificity, accuracy, and the area under the receiver operating characteristic (ROC) curve, were calculated to assess the model's predictive ability. Sensitivity, specificity, and accuracy were computed using the standard formulas. Additionally, to evaluate the discriminative ability of each feature for sample classification, we applied the univariate feature analysis method on the filtered data features, calculating the correlation between each feature and the sample class using variance analysis. Scores and corresponding *p*‐Values were calculated for each expression feature, and expression features were sorted based on their calculated *p*‐Values.

The aim was to identify the optimal subset of expression features to achieve the best prediction accuracy. We used the incremental feature selection (IFS) method to obtain the optimal subset of expression features.^[^ ^21^ ^]^ The IFS method involved constructing a feature subset in each iteration using the top i sorted expression features, followed by calculating the prediction accuracy of the feature subset for the sample using 10‐fold cross‐validation. The accuracy results were plotted against the number of features in the expression feature subset to obtain the IFS curve.

Finally, to visualize the prediction results on the optimal expression feature subset and the locally optimal expression feature subset, we used the confusion matrix of the model. The confusion matrix displayed the predicted and actual sample classifications, providing an intuitive understanding of the model's performance.

#### 2.2.10 Parallel Reaction Monitoring (PRM) Validation

PRM analysis was developed and applied to validate the differentially expressed peptides combined with clinically significant proteins as determined by WGCNA and machine learning. Each protein was quantified using two unique peptides. The synthesis of mixed samples of labeled peptides (original samples and synthetic peptide segments) was carried out using solid‐phase synthesis. Peptide synthesis occurs from the C‐terminal to the N‐terminal (amino end), whereby the first amino acid of the target peptide is linked to the solid support via a covalent bond at the C‐terminal end. Subsequently, starting from the N‐terminal of the first amino acid, the amino‐protecting group is removed, and excess activated second amino acid is reacted to extend the peptide chain. This process is repeated to achieve the desired length of the synthesized peptide chain. Finally, the peptide chain is cleaved from the resin, purified, and isolated to obtain the target peptide. PRM was performed on Orbitrap Exploris 480 (Thermo Fisher Scientific) with EASY‐nLC 1200. The resulting MS data were searched against Homo_sapiens_9606_SP_20 230 103.fasta and processed using Skyline (v.21.2). Peptide settings: enzyme was set as Trypsin [KR/P], and max missed cleavage set as 0. The peptide length was set as 7–25, and the cysteine alkylation was set as fixed modification. Transition settings: precursor charges were set as 2, 3, ion charges were set as 1, and ion types were set as b, y. The product ions were set from ion 3 to the last ion, the ion match tolerance was set as 0.02 Da.

## 3 Results

### 3.1 Study Design and Clinical Synopsis

**Figure 1:**
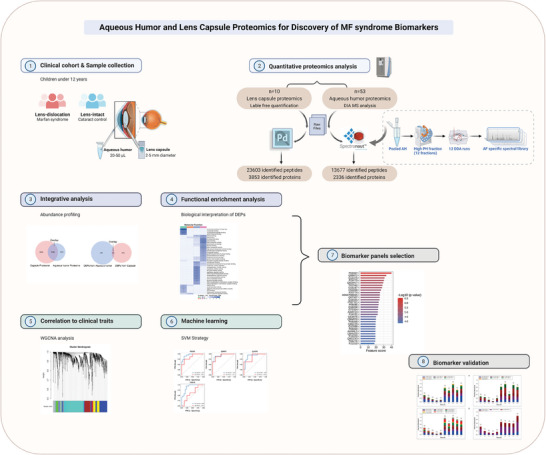
Overview of the Study Workflow.

The overall workflow of this study is shown in **Figure** **1**. During the discovery phase, 53 AH samples (27 MFS and 26 cataract controls (CC)) from 53 children and 10 lens capsule tissue samples were analyzed by LC‐MS/MS‐based DIA and label‐free quantitation. The sex, age, AL, K1, K2, Km, Cyl, axis, and ACD of cases and controls are summarized in **Table** **1**. First, we conducted differential abundance analysis and compared DEPs between two paired samples in each group. Functional enrichment analyses were used to reveal the biospecimen characterization of ectopia lentis and to further explore the underlying biological pathway of MF syndrome.

**Table 1:** Clinical characteristics of enrolled patients in the discovery phase.

Group/Characteristic/No.	MF^a)^ *N =* 27	CC^b)^ *N =* 26
Gender, Male (number)	13	14
Age (mean, SD)	6.93±2.97	5.15±3.17
AL^c)^ (mean, SD)	24.95±2.25	21.96±1.31
K1 (mean, SD)	39.28±1.41	42.99±2.40
K2 (mean, SD)	41.05±1.42	44.95±2.40
Km^d)^ (mean, SD)	40.16±1.36	43.97±2.36
Cyl^e)^ (mean, SD)	−1.77±0.75	−2.03±0.79
Axis (mean, range)	94.41 (1180)	102.75 (1179)
ACD^f)^ (mean, SD)	3.38±0.46	3.34±0.43

^a)^ Marfan syndrome;

^b)^ Cataract controls;

^c)^ Axial length;

^d)^ Mean value of K1 and K2;

^e)^ Cylinder diopter;

^f)^ Anterior chamber depth.

Next, we constructed network analyses to explore the association between protein sets with highly synergistic changes and clinically significant traits, and screened hub proteins in modulating ocular abnormities.

Finally, we applied a machine learning strategy to further screen and evaluate the features, and developed a valuable and robust biomarkers panel to aid in the early diagnosis of MF syndrome. Proteins with biological significance were externally validated using an independent cohort of 10 patients (5 MFS and 5 CC) using the PRM method.

### 3.2 AH Spectral Library Generation and its Overall Characteristics

**Figure 2:**
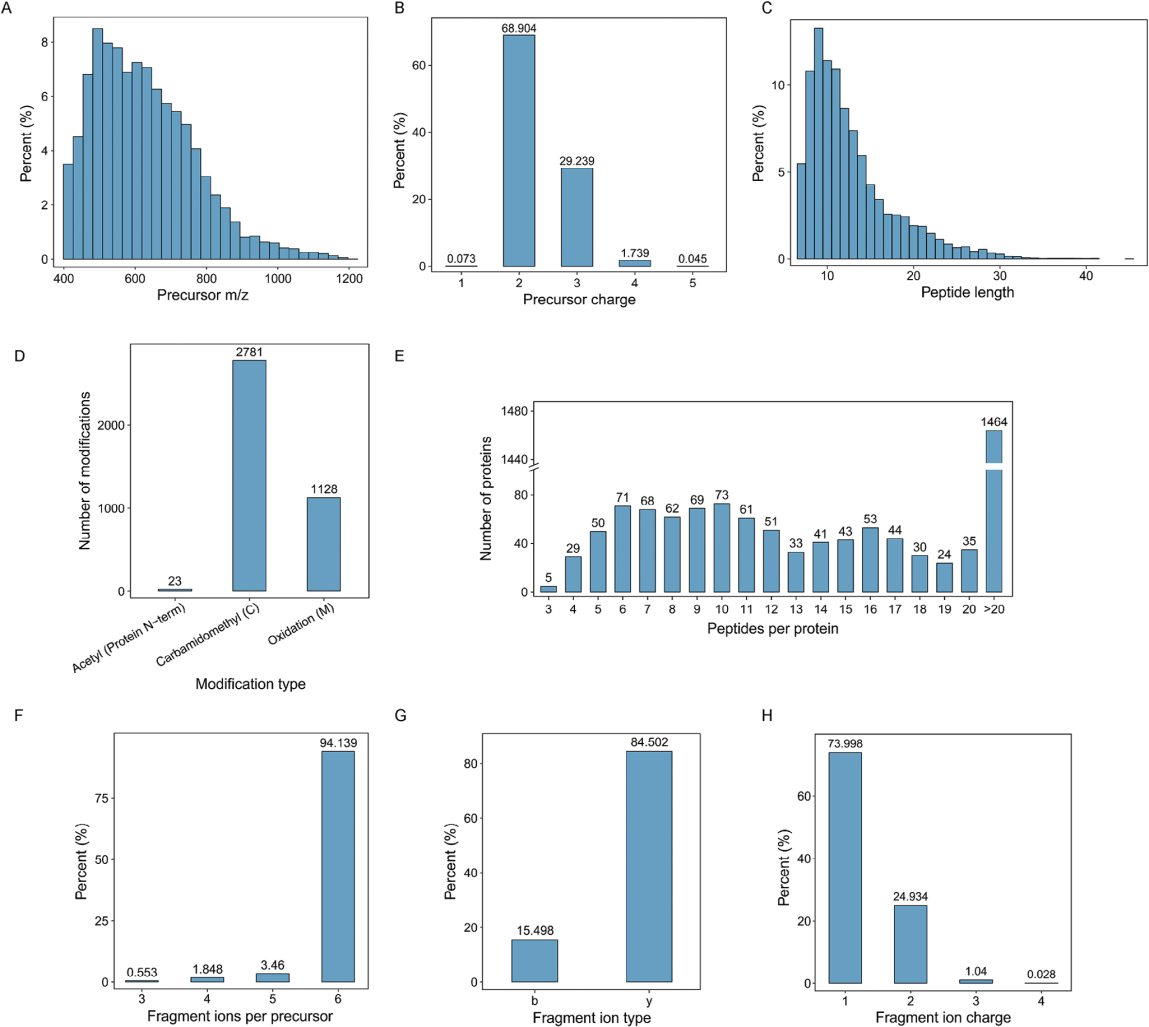
Aqueous humor (AH)‐spectral library generation and its overall characteristics. A) Distribution of precursor m/z. B) Counts of different precursor charge states. C) Distribution of peptide length. D) The number of modified peptides and distribution of different modifications. E) The number of proteotypic peptides for each protein. F) Proportion of fragment ions per precursor ion. G) Percentage of *b, y* ions. H) Proportion of different charges of fragment ions.

This study introduced an AH‐specific spectral library to support protein identification and quantification. 12‐fractioned‐pooled samples were collected by high pH reversed‐phase chromatography and acquired by DDA mode using Orbitrap Exploris 480. Our AH‐specific spectral library comprised 1,1041 precursors, 9655 peptides, 9044 proteotypic peptides, 2450 proteins, and 2306 protein groups. The investigators compared the present results with those in previously published AH proteomics studies (**Table** **2**). This new library was not only the first children‐targeted but also led to a lot more protein identification than previous ones (from 802 to 2306). The overall characteristics of the spectral library were evaluated in **Figure** **2**. The range of precursor mass covered 400–1200 m/z, and ≈81.6% of the precursors were between 450 and 800 m/z (Figure 2A). The precursors primarily showed two (68.9%) or three (29.2%) charges (Figure 2B). 91.1% of peptides were between 7 and 20 amino acids in length, consistent with the general pattern based on enzymatic and mass spectrometric fragmentation (Figure 2C). Carbamidomethyl was the most common modification found in 2781 peptides. (Figure 2D). The majority of proteins were identified with at least two proteotypic peptides, while 11464 proteins were found to have more than 20 proteotypic peptides (Figure 2E). 94.1% of peptides possessed over six fragment ions (Figure 2F). Moreover, fragments from y‐ions (84.5%) were more frequently detected than those from b‐ions (15.5%) due to basic residues at the C‐terminus digested by trypsin (Figure 2G). One (74.0%) and two (24.9%) charges comprised the majority of fragment charge distribution (Figure 2H).

**Table 2:** Comparison of recent aqueous humor (AH) proteomic studies.

Year	Reference	Disease	Methods	Instrument	FDR	Identified proteins
2020	Xue, Min et al.	Pathologic myopia	Label‐free	Triple TOF 6600	1%	583
2020	Yu, Mengxi et al.	Cataracts	Label‐free	Orbitrap Fusion Lumos Tribrid	1%	802
2021	Liu, Xiaoyan et al.	Glaucoma	DIA	Orbitrap Fusion Lumos Tribrid	1%	636
2021	Xiao, Hu et al.	Proliferative diabetic retinopathy	Tandem Mass Tag (TMT)	Orbitrap Fusion	1%	591
2022	Chen, Huan et al.	Diabetic Nephropathy	DIA	Orbitrap Fusion Tribrid	1%	692
2023	This study	Ectopia Lentis	DIA	Orbitrap Exploris 480‐FAIMS Pro	1%	2306

### 3.3 Integrative Proteomic Profiling and Functional Enrichment Analysis

A total of 2336 and 3853 proteins were identified in AH and lens capsules, respectively, with 2300 and 2938 comparable proteins (Appendix Files). Peptide length, numbers, and protein molecular weight distribution are shown in Figures S1A–C and S2A–C (Supporting Information). The intensity distribution along with its density characteristics (Figures S1D–F and S2D–F, Supporting Information) suggested that samples met quality control requirements. Protein coverage distribution in capsules is additionally displayed in Figure S2G (Supporting Information). Orthogonal partial least squares discriminant analysis (OPLS‐DA) effectively distinguished the MFS group from the CC group (Figure S1G, Supporting Information). We performed a comprehensive functional annotation of these identified proteins (Figures S1H and S2H, Supporting Information).

**Figure 3:**
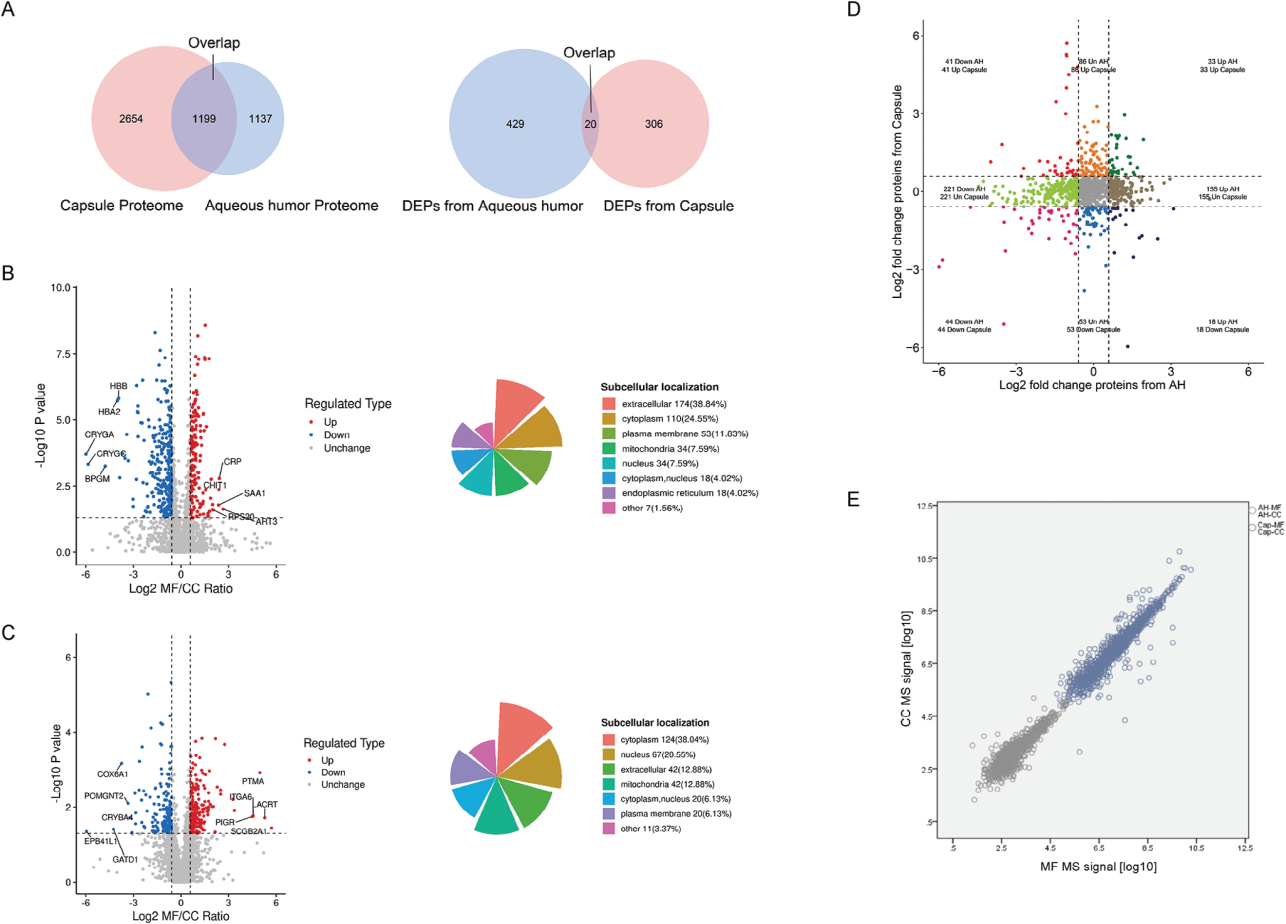
Integrative Proteomic Profiling of Children AH and Lens Capsule. A) Venn diagram showing overall proteins (Left) and DEPs (Right) identification in AH and lens capsule. B) Volcano plots (Left) and polar area diagram (Right) showing DEPs and their subcellular localization in AH of MFS and CC patients. C) Volcano plots (Left) and polar area diagram (Right) showing DEPs and their subcellular localization in lens capsule of MFS and CC patients. D) Nine‐quadrant chart showing the distribution of overlapping proteins in AH and capsules with Log2 fold change. E) AH–capsule proteome abundance map showing the median protein intensity (assessed by MS intensity) of overlapping proteins. AH, aqueous humor; CC, cataract controls; DEPs, differentially expressed proteins with a 1.5‐fold change and a threshold‐adjusted *p*‐Value of less than 0.05; MFS, Marfan syndrome.

We then processed comparable analysis showing significantly and differentially altered proteins between MFS and CC. As the volcano plots in **Figure** **3**B show, by setting a cutoff value of a 1.5‐FC and a threshold adjusted *p*‐Value of less than 0.05, we identified 449 DEPs in AH, specifically, 155 upregulated and 294 downregulated proteins. Of these, 38.84% were from the extracellular space, which is consistent with the physiological properties of AH. In addition, the comparison showed 326 DEPs (38.04% cytoplasmic subcellular localization) in lens capsules, with 178 upregulated and 148 downregulated proteins (Figure 3C). Interestingly, only 20 DEPs were commonly differentially abundant between AH and capsule proteome (Figure 3A, right), which is a much smaller number compared to the overall number of commonly detected proteins (Figure 3A, left). Figure 3D was used to compare the overlapping proteins in detail, by analyzing their Log2 FC. Figure 3E revealed a linear correlation between their relative abundance rank in MFS and CC. It is noteworthy that these proteins were generally detected at much lower levels in AH than in capsules, presumably reflecting tissue leakage.

**Figure 4:**
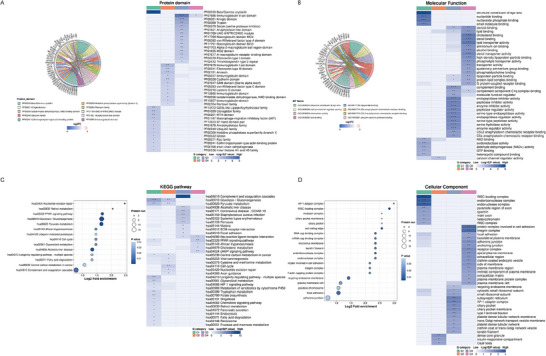
Key Pathway, Function, and Proteins Characterized in MFS and CC Patients. A) Enrichment chord graph of DEPs domain in AH (Left) and its four sets of hierarchical clustering analysis (Right). B) Enrichment chord graph of DEPs molecular function in AH (Left) and its four sets of hierarchical clustering analysis (Right). C) Enrichment bubble plot of DEPs KEGG pathway in AH (Left) and its four sets of hierarchical clustering analysis (Right). D) Enrichment bubble plot of DEPs cellular component in lens capsule (Left) and its four sets of hierarchical clustering analysis (Right). AH, aqueous humor; CC, cataract controls; DEPs, differentially expressed proteins with a 1.5‐fold change and a threshold‐adjusted *p*‐value of < 0.05; KEGG, Kyoto Encyclopedia of Genes and Genomes; MFS, Marfan syndrome.

To further investigate the biological function of DEPs in MF syndrome, we performed comprehensive functional enrichment analyses. Based on the 449 DEPs detected in AH (**Figure** **4**A), Beta/Gamma crystallins, which constitute major components of lens proteins, were the most significantly enriched protein domain, indicating that they may be leaked or secreted into the AH. Those DEPs were also found to be involved in several major molecular functions, namely, structural constituent of the eye lens, lipid binding, calcium channel regulator activity, ubiquitin‐specific protease binding, and C5a and C5L2 anaphylatoxin chemotactic receptor binding, as annotated in Figure 4B. Additionally, pathway annotation and enrichment analysis revealed that upregulated proteins were overrepresented in complement and coagulation cascades and the PPAR signaling pathway, while the downregulated proteins were enriched in glycolysis/gluconeogenesis and pyruvate metabolism (Figure 4C).

DEPs in lens tissue were also analyzed to explore mechanisms underlying ectopia lentis by mapping to the cellular component. The obtained results showed dysregulation of proteins involved in focal adhesion, RISC‐loading complex, and cell trailing edge (Figure 4D).

For further hierarchical clustering based on DEPs functional classification, we first divided them into 4 categories according to their differential expression folds, called Q1 to Q4 (< 0.5, 0.5‐0.667, 1.5‐2.0, > 2.0, respectively). Then, for each Q group, the molecular function, cellular component, and the KEGG pathway were enriched separately, and cluster analysis was performed to find the correlation of protein functions with differential expression folds in the comparison groups. The corresponding enrichment‐based clustering are displayed in Figure 4A–D, right. Data suggested that DEPs in AH linked to the complement and coagulation cascades were highlighted in the Q3 cluster, while glycolysis/gluconeogenesis and pyruvate metabolism‐related ones were in the Q1 cluster.

### 3.4 Identification of Clinically Significant Protein Modules

**Figure 5:**
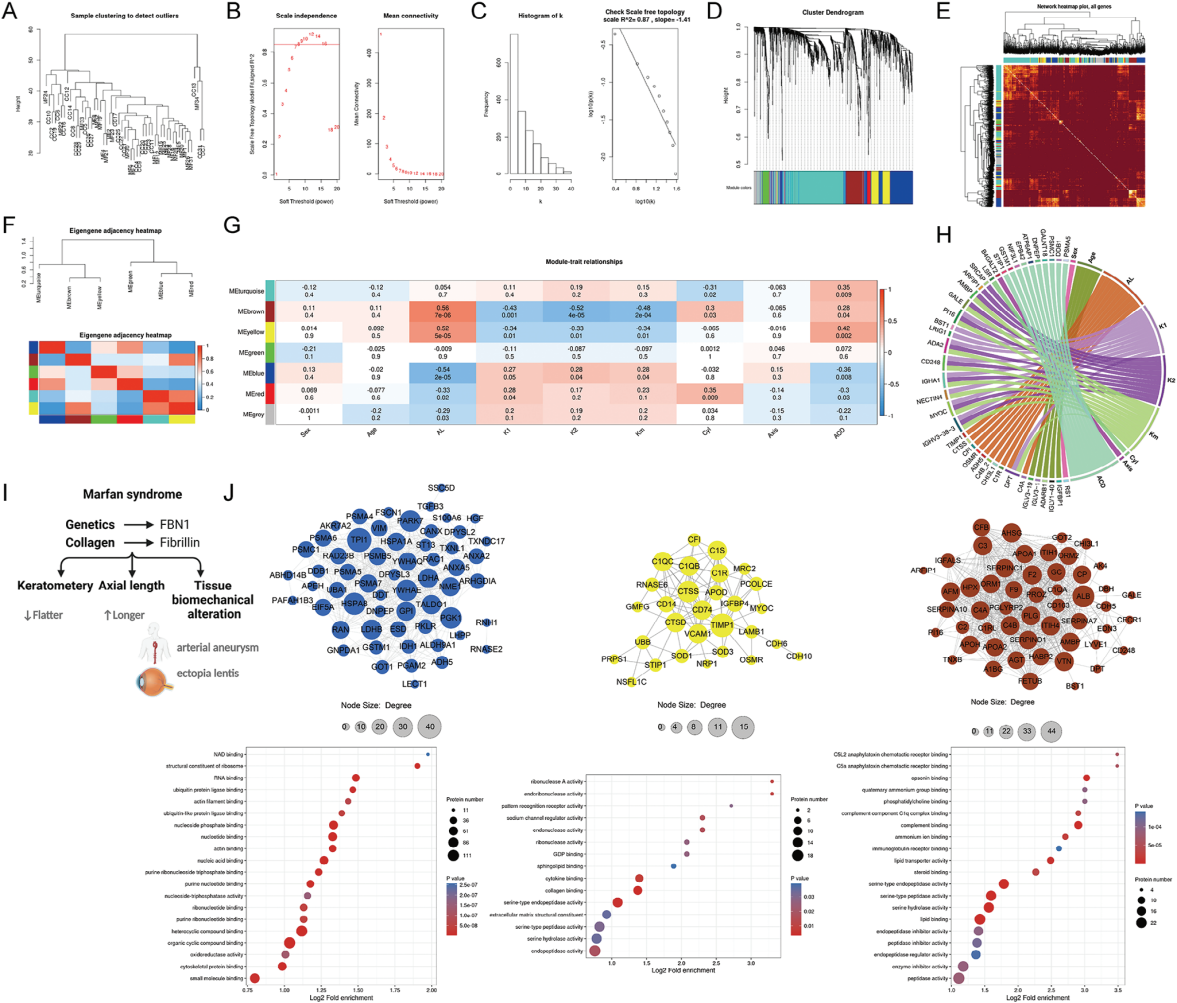
Identification of clinically significant protein modules. A) Sample clustering for outlier detection. To identify any sample outliers in the AH dataset, average linkage hierarchical clustering was conducted. Results indicate that there were no sample outliers. B) Power transformation analysis and assessment of scale‐free topology criteria. We tested powers ranging from 1 to 20 to determine the optimal transformation. The red line (0.85) represents the scale‐free topology criterion; values greater than this indicate satisfactory adherence to the criterion. We observed that increasing values of β lead to decreased mean connectivity, suggesting that the network comprises many proteins with few connections. Moreover, the decay of mean connectivity follows an inverse power law, further supporting the scale‐free topology assumption. C) Scale‐free topology checking. Distribution of nodes with the degree of connection, k. (Left) and correlation between log (k) and log [P(k)] (Right). D) Seven distinct modules of highly co‐expressed proteins were identified based on the hierarchical clustering dendrogram. E) TOM for distinctive modules; red shades mark higher topology overlap shared between the correlated proteins in the network. F) Eigenprotein dendrogram and heat map where red and blue represent high and low correlations of the eigenproteins, respectively. G) The module‐trait correlation plot according to the clustering dendrogram of module eigengenes. The heat map illustrated positive correlations as red and negative correlations as blue. The values in each grid represented the correlation coefficient and the corresponding *p*‐value between the module and clinical traits, with the second row of values indicating the significance level of the correlation. H) Chord graph showing the correlation between ocular features and the top 10 proteins with the highest GS value in each feature. I) Clinicopathological signatures of MFS focused in this study. J) PPI network (Up) and molecular function enrichment analysis (Down) of proteins in three core modules, MEbrown, MEyellow, and MEblue. AH, aqueous humor; GS, gene significance; MFS, Marfan syndrome; PPI, protein‐protein interaction; TOM, topological overlap map.

The hierarchical clustering analysis revealed close relationships among samples, indicating that there was no need to exclude any samples and that all samples could be used for WGCNA (**Figure** **5**A). A power value (β) of 8 was selected as the soft threshold to construct the adjacency matrix, and the resulting network based on β = 8 exhibited a scale‐free topology (Figure 5B,C). Using hierarchical clustering and dynamic tree‐cutting methods, a total of seven distinct co‐expressed modules were obtained, each represented by a different color, i.e., turquoise, brown, yellow, green, blue, red, and grey modules, with grey indicating genes that could not be assigned to any module (Figure 5D). Figure 5E displayed the modules’ topological overlap map (TOM). Distinct modules were differentiated based on the clustering dendrogram of MEs and module‐module associations are depicted in an eigengenes adjacency heat map in Figure 2F.

Furthermore, correlations between modules and phenotype data were analyzed and displayed using a heat map in Figure 5G. MEbrown, MEyellow, and MEblue exhibited significant correlations with Km (correlation coefficient *r =* −0.48, −0.34, 0.28, respectively, *p <* 0.05). Strong associations with Cyl were observed for MEturquoise, MEbrown, and MEred (*r =* −0.31, 0.3, 0.35, respectively, *p <* 0.05). MEyellow and MEblue were highly correlated with ACD (*r =* 0.42, −0.36, respectively, *p <* 0.01). Moreover, a significant positive correlation with AL was observed for MEbrown and MEyellow (*r =* 0.56, 0.52, respectively, *p <* 0.001), whereas a significant negative correlation was found for MEblue and MEred (*r =* −0.54, −0.33, respectively, *p <* 0.001).

To narrow the scope of key proteins, we filtered for proteins with absolute values of GS greater than 0.05. Higher GS values indicate a more pronounced correlation between proteins and clinical traits. Figure 5H displays the correlation between ocular features and the top 10 proteins with the highest absolute value in each feature. The three resulting crucial modules, MEbrown, MEyellow, and MEblue are predominately linked with AL and corneal keratometry (K1, K2, Km), and correspondingly, longer ALs and flatter corneas are two distinctive features of patients with MFS (Figure 5I). Their biological continuity is well exhibited among proteins in AL‐or‐keratometry‐related modules by plotting the hub proteins in the PPI network (Figure 5J, up). These modules are enriched in collagen, complement, cytokine and opsonin binding, and ribosomal structural molecular function (Figure 5J, down).

### 3.5 Machine‐learning–based Selection of Biomarker Panels

**Figure 6:**
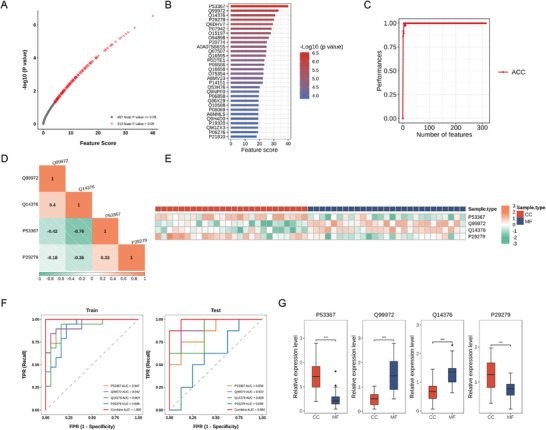
Machine‐learning‐based Identification of Biomarker Panels Signatures. A) Evaluation of feature discrimination ability and visualization of sorted expression features: We used univariate feature analysis to sort the expression features based on their score and *p*‐value. The *x*‐axis represents the score of each expression feature, while the *y*‐axis indicates the ‐log10‐transformed *p*‐value of the corresponding feature. The expression features with *p*‐values <0.05 are highlighted in red, while those with *p*‐Values > 0.05 are shown in gray. B) The bar plot displays the top 30 features with the highest scores, and the colors of the bars are filled based on the ‐log10 *p*‐value of the corresponding feature. C) IFS curve was generated to identify the optimal subset of expression features for predicting sample class with the highest accuracy using the incremental feature selection method. The curve shows the relationship between the number of features and the corresponding accuracy achieved through 10‐fold cross‐validation. D) Heat map showing the Pearson correlation coefficients of expression data for the selected features across different sample classes, indicating low correlation and no feature redundancy in the optimal expression feature subset. E) Heat map showing the expression levels of the proteins in the optimal feature subset across all samples. F) ROC curves showing the ability of selected features to accurately classify samples in the train and test dataset. G) Box plot illustrating the distribution differences of protein expression among different sample groups. IFS, Incremental Feature Selection; ROC, Receiver Operator Characteristic.

We applied a machine‐learning approach to detect protein subsets that could potentially function as AH‐biopsy signatures and integrated them into unified predictors for accurate discrimination of patients at risk. Specifically, we assessed quantified proteins as input features for machine learning and determined the most relevant ones using the SVM algorithm.^[^ ^20^ ^]^ To this end, we performed a univariate feature analysis to measure the importance of each protein in discriminating between two classes of patients (MFS or CC) and subsequently ranked them based on their scores and *p*‐Values, as shown in **Figure** **6**A. The top 30 ranked proteins are displayed in Figure 6B. To obtain the optimal subset of candidates, we employed the IFS method (Figure 6C). We assessed the prediction performance of selected proteins using AUC. A set of candidates that showed the highest AUC was selected as being the most relevant features. Using these proteins as features, the best diagnostic model was generated. We found that the four top‐ranked features, mapping to the four respective distinct gene products (CCN2, ARFIP1, GALE, and MYOC) showed the best performance in discriminating patients. Heat maps depicting their Pearson correlation coefficients and quantitative levels were created, with the data suggesting that our model had no redundancy (Figure 6D,E). In the test set, the diagnostic model showed a combined AUC of 0.98 (Figure 6F). Box plots presented their relative expression variations taken from all samples together (Figure 6G).

### 3.6 Selected Biomarkers Validation and Clinical Correlation

**Figure 7:**
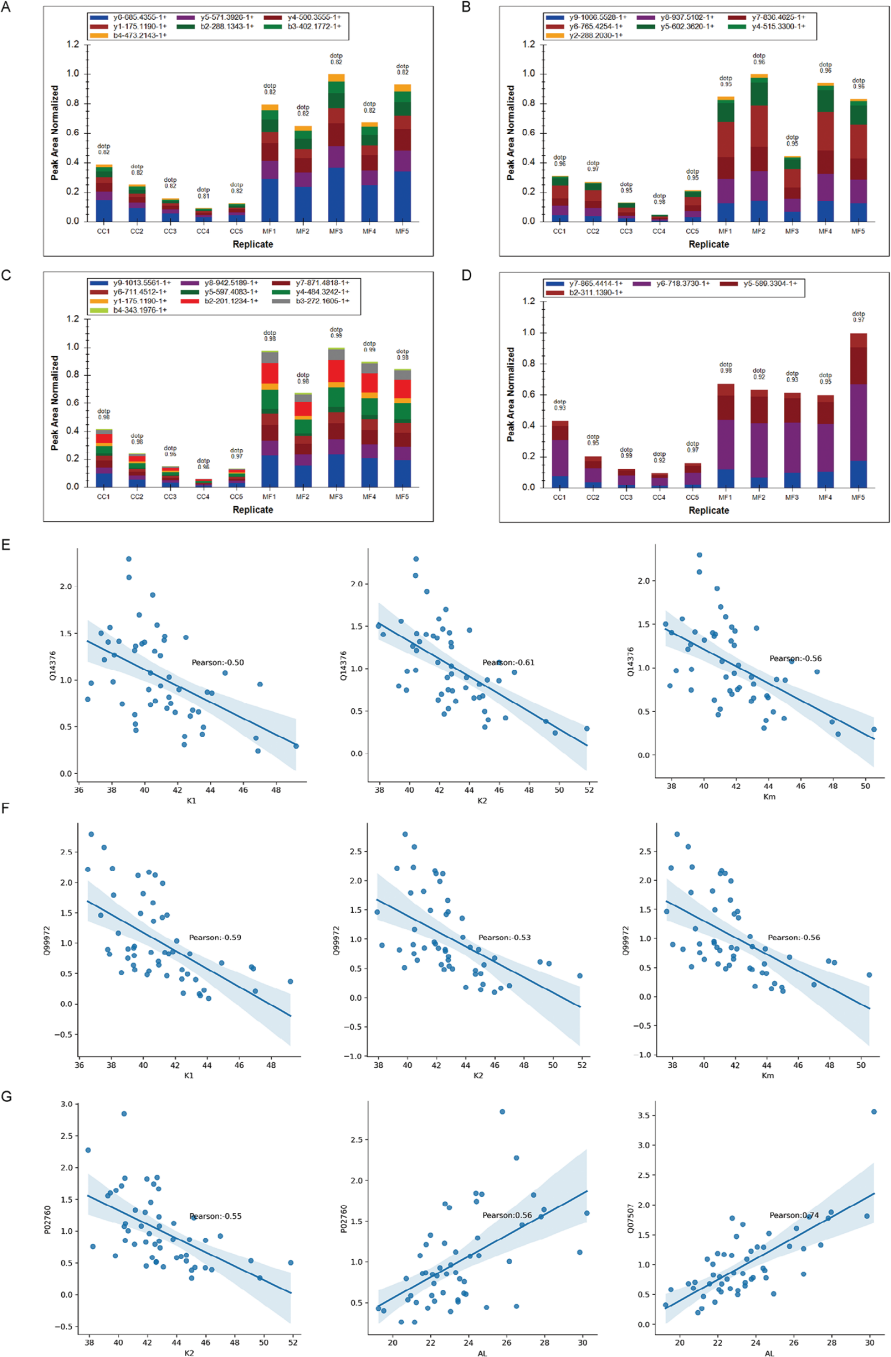
Biomarker Expression Plots via PRM Validation and Clinical Correlation. A) Distribution of fragment ion peak areas of peptide TWNAVLLR (corresponding to protein Q14376) in the validation cohort. B) Distribution of fragment ion peak areas of peptide ELETAYSNLLR (corresponding to protein Q99972) in the validation cohort. C) Distribution of fragment ion peak areas of peptide TVAACNLPIVR (corresponding to protein P02760) in the validation cohort. D) Distribution of fragment ion peak areas of peptide YFESVLDR (corresponding to protein Q07507) in the validation cohort. E‐G) Pearson correlation analysis between clinical indicators and protein biomarkers: E: Q14376; F: Q99972; G: P02760 (Left, Middle) and Q07507 (Right). PRM, Parallel Reaction Monitoring.

PRM validation was performed on candidate proteins in an independent cohort. Among the sixteen top‐ranked peptides selected as the most relevant biomarker signatures for predicting MF syndrome in children, twelve proteins were identified from WGCNA due to their significant clinical values, while the other four proteins were derived from machine learning. We have successfully quantified 15 out of the 16 target proteins. We were able to identify only one peptide for some of the proteins due to sensitivity limitations. Notably, four proteins (Q14376, Q99972, P02760, Q07507; Gene name, GALE (UDP‐galactose‐4‐epimerase), MYOC (Myocilin), AMBP (Alpha‐1‐microglobulin/bikunin precursor), DPT (Dermatopontin), respectively) were confirmed to be the most robust in this independent sample set, as evidenced by the distribution of the ion peak area of their unique peptides (TWNAVLLR, ELETAYSNLLR, TVAACNLPIVR, YFESVLDR, respectively) in **Figure** **7**A–D, which showed significant differences (*p*‐Value < 0.005 or less). Furthermore, the correlation between the level of these potential biomarkers and clinical signatures was analyzed, as shown in Figure 7E–G. The results indicated that Q14376, Q99972, and P02760 were negatively correlated with keratometry (K1, K2, Km), while P02760 and Q07507 positively correlated with AL. These findings further corroborated our proteomic discoveries regarding two distinctive features of patients with MFS in the discovery cohort.

## 4 Discussion

Here we set out to employ cutting‐edge MS technology to reveal the proteomic profile of AH proteins in children, significantly improving the identification depth and providing novel insights into the complexity of the AH proteome. Integrated analysis of the crystalline lens anterior capsule and AH deepened our understanding of the molecular mechanisms underlying MFS. We identified protein modules strongly correlated with MFS clinical features through WGCNA and applied machine‐learning techniques to select highly robust biomarkers. Validation of the biomarker panel using advanced PRM underscores its potential utility in clinical settings.

Significant strides have been made in uncovering the heritable risk of MFS through genetics, but the impact of these genetic findings on the biological pathways that underpin MFS pathophysiology remains unclear.^[^ ^3^, ^22^ ^]^ While our team has made considerable contributions to investigating the genetic basis of ectopia lentis,^[^ ^23^, ^24^, ^25^, ^26^ ^]^ the ultimate effectors of MFS genetic risk are often the proteins and the metabolic pathways that they regulate. In recent years, proteomics has emerged as a powerful tool for elucidating the molecular mechanisms that drive various diseases and identifying key biomarkers for diagnosis and prognosis.^[^ ^27^ ^]^ Proteomics research is gaining momentum among scientists due to the rapid advances in MS and sample preparation techniques.^[^ ^28^, ^29^ ^]^

DIA is a MS‐based technique that has shown promise in enabling the simultaneous and reproducible quantification of hundreds or thousands of peptides across multiple samples. DIA offers several advantages over other approaches, including unbiased and comprehensive proteomic coverage, the detection and quantification of low‐abundance peptides, and the ability to measure multiple samples in a single experiment.^[^ ^30^, ^31^ ^]^ Unlike conventional proteomics, DIA is less susceptible to the influence of high‐abundance proteins, eliminating the necessity for affinity‐based depletion methods that may introduce bias or incur additional costs, particularly in samples such as plasma that are rich in high‐abundance proteins.^[^ ^32^ ^]^

AH is an important fluid that plays a vital role in maintaining the ocular environment by providing nutrients and oxygen to the cornea and lens, which lack their own blood supply.^[^ ^33^ ^]^ In addition, AH assists in the removal of waste products from the eye, thereby contributing to the overall health of the eye.^[^ ^33^ ^]^ This clear, watery fluid located in the anterior and posterior chambers of the eye is critical for the proper functioning of the visual system. The unique properties of AH make it an attractive target for proteomics studies aimed at identifying new biomarkers and therapeutic targets for ocular diseases.^[^ ^34^, ^35^, ^36^ ^]^ The study of AH proteomics is an emerging field that has yet to be fully explored, with inconsistent depths of identification observed across different studies.^[^ ^17^, ^35^, ^37^ ^]^ However, AH has been shown to share similar protein compositions with plasma,^[^ ^38^ ^]^ suggesting the potential to apply established plasma proteomic methods to AH. Despite the low protein concentration in AH (0.02% in AH versus 10% in plasma),^[^ ^34^, ^38^ ^]^ the depth of identification in previous studies might have been limited. In this study, we identified over 2000 proteins in AH, which is significantly higher than previous reports, due to the use of DIA and the emerging ion mobility MS (IM‐MS) technology.^[^ ^39^ ^]^ The combination of differential mobility spectrometry (DMS) with IM‐MS, specifically the FAIMS‐IM approach, was employed to enhance the sensitivity and specificity of protein identification.^[^ ^18^, ^40^, ^41^ ^]^ The FAIMS device acts as a filter that selectively transmits ions based on their mobility characteristics, allowing for the separation of ions with similar mass‐to‐charge ratios but different mobilities.^[^ ^18^, ^40^ ^]^ We utilized the state‐of‐the‐art Orbitrap Exploris 480 mass spectrometer equipped with a FAIMS Pro Interface to obtain DIA data that leveraged DMS techniques and achieved higher detection sensitivity. Our approach enabled the detection of low‐abundance proteins in AH while reducing batch effects and ensuring experimental parallelism.

The existing diagnosis criteria for MFS^[^ ^42^ ^]^ based on features of the ocular, skeletal, and thoracic systems have limited accuracy in the early disease stages, thus severely reducing opportunities for timely disease detection and intervention. Asymptomatic aortic root aneurysms, in cases where MFS is left undiagnosed due to the lack of recognition of skeletal or ophthalmic manifestations, can eventually progress to acute aortic dissections, which itself serve as another crucial diagnostic feature.^[^ ^3^, ^42^ ^]^ In fact, MFS may go undiagnosed until an individual suffers from an acute aortic dissection, thus highlighting the importance of genetic testing for FBN1 variants.^[^ ^43^, ^44^ ^]^ It is worth noting that the criteria used to diagnose MFS according to Ghent II criteria,^[^ ^42^ ^]^ which take into account skeletal growth abnormalities such as pectus deformities and scoliosis, may not become fully apparent until an individual reaches skeletal maturity.^[^ ^3^ ^]^ It is also worth noting that MFS patients of Hispanic and Asian origins have been observed to present with fewer skeletal symptoms, yet still display similar ophthalmic and thoracic features as Europeans who have MFS.^[^ ^45^, ^46^ ^]^ As a result, these patients may be less likely to receive a referral for MFS evaluation based on skeletal symptoms. Therefore, it is imperative to develop broader diagnostic approaches that target at‐risk populations, including those with a history of ectopia lentis, particularly in children.

In recent years, the heightened importance placed on early education by parents, coupled with the popularity of vision screening, has resulted in a noticeable upsurge in the number of ectopia lentis cases presented to ophthalmology clinics. Consequently, ophthalmologists are often the first to detect potential patients with MF syndrome by identifying children with reduced visual acuity. Were a proximal body fluid (i.e., AH) protein biomarker to be identified, the diagnosis of this disease could be ascertained in a timely manner. Such an advance would enable ophthalmologists to inform parents of the child of the need to carefully monitor the development of the cardiovascular and skeletal systems over time, ultimately leading to early diagnosis, intervention in life‐threatening comorbidities such as aortic dilatation, and, eventually, preservation of the patient's life. However, no relevant studies have reported the discovery of such biomarkers in samples similar to those discussed in this paper.

Recent studies have highlighted the importance of considering AL and corneal curvature in the ophthalmic evaluation of suspected and diagnosed cases of MFS.^[^ ^47^ ^]^ While these parameters are not currently included as diagnostic criteria in the revised Ghent‐2 nosology, the Marfan Eye Consortium of Chicago recommends that patients with longer AL and flatter corneas should be considered as potential MFS cases.^[^ ^6^ ^]^ In a study by Martin Heur et al., there was a significant difference in corneal curvature values between MFS and control patients, with values less than 42 D potentially serving as a clinical diagnostic criterion for MFS.^[^ ^48^ ^]^ These findings emphasize the importance of ophthalmologists being aware of the potential diagnostic value of AL and corneal keratometry in the diagnosis of MF syndrome.^[^ ^6^, ^47^, ^48^ ^]^ In this context, we employed the WGCNA method to establish a gene co‐expression network linked to the clinical characteristics of MFS. Surprisingly, we discovered that the protein modules correlated with AL and corneal curvature had significantly higher GS scores and were functionally linked with each other.

Recent research on aortic aneurysms in MFS indicates that the progression of aneurysms may involve extracellular matrix remodeling, cell adhesion, and complement activation.^[^ ^49^, ^50^, ^51^, ^52^ ^]^ In 2022, Stijntje Hibender et al. detected a complement gene C1R variant linked to aortic comorbidity through whole‐genome sequencing (WGS) on a Marfan family.^[^ ^51^ ^]^ A single‐cell RNA sequencing workflow applied to aortic aneurysm samples collected in Fbn1C1041G/+ (MFS) mice and controls reported a cluster of altered genes involved in extracellular matrix modulation, collagen synthesis, and adhesion.^[^ ^52^ ^]^ In our study, through functional enrichment analysis of DEPs in AH and lens tissue, we discovered that MFS patients with lens dislocation exhibit significant dysregulation of complement and coagulation‐related functions in AH. In contrast, the DEPs identified in the lens tissue were primarily associated with cell adhesion. Further analysis using WGCNA enabled us to examine the clinical relevance of all quantified AH proteins rather than focusing on DEPs. Our results demonstrated that the MEbrown and MEyellow modules, which were highly correlated with key clinical indicators including AL and corneal curvature as discussed above, were also enriched for complement, cytokine, opsonin, and collagen binding. Notably, highly interactive proteins in MEbrown and MEyellow, including C1R, C1QB, C4A, C4B, and CD74, were associated with the complement system, while PLG, SERPIND1, SERPINC1, PROZ, F2, F9 were associated with coagulation, and VCAM1 with cell adhesion. Our findings suggest that complement and coagulation‐related proteins may play critical roles in the pathogenesis of MFS‐related ocular complications. To the best of our knowledge, this is the first study to construct a co‐expression network associated with the clinical features of MFS. This innovative and comprehensive approach provides a new avenue for discovering potential biomarkers, identifying critical genes, and developing more effective diagnostic methods for MFS. It is noteworthy that four proteins (GALE, MYOC, AMBP, and DPT) met our validation criteria for PRM and are all associated with keratometry or AL. DPT, which plays a role in collagen fibrillogenesis, may contribute to connective tissue disorders, as previously reported.^[^ ^53^ ^]^ Therefore, the potential involvement of DPT in MFS pathogenesis is a plausible hypothesis. This indicates that targeted proteomics analysis in clinically relevant tissue proximal fluids can identify peptide signatures that predict the onset of MFS before the manifestation of cardiovascular symptoms, thus facilitating early diagnosis.

In conclusion, our study has provided compelling evidence that the AH, a crucial ocular biofluid, contains a significant amount of unidentified proteins that can serve as biomarkers for ectopia lentis in MF syndrome. Utilizing network analysis and machine learning techniques can further assist in screening and assessing the biomarkers with clinical relevance, which, when combined with PRM validation, can lead to the development of a robust and valuable panel of biomarkers for early detection of MF syndrome. Our AH proteomics workflow has been streamlined by the introduction of an AH‐specific spectral library, which can be easily scaled up for use in larger and more powerful cohorts. Future studies should incorporate longitudinal data to validate the increased levels of GALE, MYOC, AMBP, and DPT in MFS patients as potential indicators of early‐stage disease and also to explore new biomarkers for other ocular system syndromes.

## Conflict of Interest

The authors declare no conflict of interest.

## Supporting information

Supporting Information

Supporting Table 1

Supporting Table 2

## Data Availability

The data that support the findings of this study are available on request from the corresponding author. The data are not publicly available due to privacy or ethical restrictions.

## References

[1] L. Pollock , A. Ridout , J. Teh , C. Nnadi , D. Stavroulias , A. Pitcher , E. Blair , P. Wordsworth , T. Vincent , Curr. Rheumatol. Rep. 2021, 23, 81. 34825999 10.1007/s11926-021-01045-3 PMC8626407

[2] S. Coelho , A. Almeida , Rev Port Cardiol (Engl Ed) 2020, 39, 215. 32439107 10.1016/j.repc.2019.09.008

[3] D. Milewicz , A. Braverman , J. De Backer , S. Morris , C. Boileau , I. Maumenee , G. Jondeau , A. Evangelista , R. Pyeritz , Nat. Rev. Dis. Primers 2021, 7, 64. 34475413 10.1038/s41572-021-00298-7 PMC9261969

[4] C. Nienaber , Y. Kodolitsch , Cardiol. Rev. 1999, 7, 332. 11208245 10.1097/00045415-199911000-00011

[5] R. Pyeritz , Genet Med 2019, 21, 1683. 30573797 10.1038/s41436-018-0399-4

[6] M. Kinori , S. Wehrli , I. Kassem , N. Azar , I. Maumenee , M. Mets , Am. J. Ophthalmol. 2017, 177, 144. 28257833 10.1016/j.ajo.2017.02.022 PMC5648325

[7] T. Chen , M. Deng , M. Zhang , J. Chen , Z. Chen , Y. Jiang , Sci. Rep. 2021, 11, 2994. 33542371 10.1038/s41598-021-82586-6 PMC7862488

[8] D. Salchow , P. Gehle , Eur. J. Ophthalmol. 2019, 29, 38. 29587526 10.1177/1120672118761333

[9] N. Zadeh , J. Bernstein , A. Niemi , S. Dugan , A. Kwan , D. Liang , J. Hyland , H. Hoyme , L. Hudgins , M. Manning , Am J Med Genet A 2011, 155a, 2661. 21932315 10.1002/ajmg.a.34245

[10] C. Barbas‐Bernardos , E. Armitage , A. García , S. Mérida , A. Navea , F. Bosch‐Morell , C. Barbas , J. Pharm. Biomed. Anal. 2016, 127, 18. 27036676 10.1016/j.jpba.2016.03.032

[11] K. Pietrowska , D. Dmuchowska , P. Krasnicki , A. Bujalska , P. Samczuk , E. Parfieniuk , T. Kowalczyk , M. Wojnar , Z. Mariak , A. Kretowski , M. Ciborowski , Electrophoresis 2018, 39, 1233. 29292830 10.1002/elps.201700411

[12] A. Danise , P. Cinque , S. Vergani , M. Candino , S. Racca , A. De Bona , R. Novati , A. Castagna , A. Lazzarin , Clin. Infect. Dis. 1997, 24, 1100. 9195064 10.1086/513625

[13] T. Freddo , Prog Retin Eye Res 2013, 32, 181. 23128417 10.1016/j.preteyeres.2012.10.004 PMC3544162

[14] D. Nusinow , J. Szpyt , M. Ghandi , C. Rose , E. Mcdonald , M. Kalocsay , J. Jané‐Valbuena , E. Gelfand , D. Schweppe , M. Jedrychowski , J. Golji , D. Porter , T. Rejtar , Y. Wang , G. Kryukov , F. Stegmeier , B. Erickson , L. Garraway , W. Sellers , S. Gygi , Cell 2020, 180, 387. 31978347 10.1016/j.cell.2019.12.023 PMC7339254

[15] R. Rotello , T. Veenstra , Curr. Protein Pept. Sci. 2021, 22, 121. 32957902 10.2174/1389203721666200921153513

[16] L. Agnifili , D. Pieragostino , A. Mastropasqua , V. Fasanella , L. Brescia , G. Tosi , P. Sacchetta , L. Mastropasqua , Prog Brain Res 2015, 221, 1. 26518070 10.1016/bs.pbr.2015.05.006

[17] R. Semba , J. Enghild , V. Venkatraman , T. Dyrlund , J. Eyk , Proteomics 2013, 13, 2500. 23749747 10.1002/pmic.201300075 PMC3978387

[18] D. Bekker‐Jensen , A. Martínez‐Val , S. Steigerwald , P. Rüther , K. Fort , T. Arrey , A. Harder , A. Makarov , J. Olsen , Mol. Cell. Proteomics 2020, 19, 716. 32051234 10.1074/mcp.TIR119.001906 PMC7124470

[19] L. Beretta , A. Santaniello , BMC Med Inform Decis Mak 2016, 16, 74. 27454392 10.1186/s12911-016-0318-z PMC4959387

[20] D. Bzdok , M. Krzywinski , N. Altman , Nat. Methods 2018, 15, 5. 30100821 10.1038/nmeth.4551 PMC6082635

[21] S. Gao , P. Wang , Y. Feng , X. Xie , M. Duan , Y. Fan , S. Liu , L. Huang , F. Zhou , Comput. Biol. Med. 2021, 133, 104405. 33930763 10.1016/j.compbiomed.2021.104405

[22] S. Zeigler , B. Sloan , J. Jones , Adv. Exp. Med. Biol. 2021, 1348, 185. 34807420 10.1007/978-3-030-80614-9_8 PMC8915437

[23] Z. Chen , W. Jia , Y. Jiang , Front Genet 2022, 13, 943083. 36176293 10.3389/fgene.2022.943083 PMC9514320

[24] Z. Chen , T. Chen , M. Zhang , J. Chen , L. Lan , M. Deng , J. Zheng , Y. Jiang , Hum Mutat 2021, 42, 1637. 34550612 10.1002/humu.24283

[25] T. Chen , Z. Chen , M. Zhang , J. Chen , M. Deng , J. Zheng , L. Lan , Y. Jiang , Am. J. Ophthalmol. 2022, 237, 278. 34818515 10.1016/j.ajo.2021.11.014

[26] Z. Chen , T. Chen , M. Zhang , J. Chen , M. Deng , J. Zheng , L. Lan , Y. Jiang , Br. J. Ophthalmol. 2022, 106, 1655. 34281902 10.1136/bjophthalmol-2021-319084 PMC9685704

[27] L. Dayon , O. Cominetti , M. Affolter , Expert Rev. Proteomics 2022, 19, 131. 35466824 10.1080/14789450.2022.2070477

[28] L. F. Vistain , S. Tay , Trends Biochem. Sci. 2021, 46, 661. 33653632 10.1016/j.tibs.2021.01.013 PMC11697639

[29] X.u Li , W. Wang , J. Chen , Sci China Life Sci 2017, 60, 1093. 29039124 10.1007/s11427-017-9175-2

[30] C. Ludwig , L. Gillet , G. Rosenberger , S. Amon , B. Collins , R. Aebersold , Mol Syst Biol 2018, 14, e8126. 30104418 10.15252/msb.20178126 PMC6088389

[31] X. Zhong , D. Frost , Q. Yu , M. Li , T. Gu , L. Li , Anal. Chem. 2020, 92, 11119. 32649829 10.1021/acs.analchem.0c01136 PMC7438256

[32] H. Fang , D. Greening , Methods Mol Biol 2023, 2628, 93. 36781781 10.1007/978-1-0716-2978-9_7

[33] K. Pietrowska , D. Dmuchowska , P. Krasnicki , Z. Mariak , A. Kretowski , M. Ciborowski , J. Pharm. Biomed. Anal. 2018, 159, 23. 29980016 10.1016/j.jpba.2018.06.049

[34] H. Youngblood , R. Robinson , A. Sharma , S. Sharma , Int. J. Mol. Sci. 2019, 20, 4755. 31557880 10.3390/ijms20194755 PMC6801709

[35] S. Funke , N. Perumal , K. Bell , N. Pfeiffer , F. H. Grus , Expert Rev. Proteomics 2017, 14, 311. 28271721 10.1080/14789450.2017.1298448

[36] H. Chen , T. Wang , E. Wang , N. Li , H. Min , Oxid Med Cell Longev 2022, 2022, 5945828. 36211816 10.1155/2022/5945828 PMC9537621

[37] W. Hubens , R. Mohren , I. Liesenborghs , L. Eijssen , W. Ramdas , C. Webers , T. Gorgels , Exp. Eye Res. 2020, 197, 108077. 32470343 10.1016/j.exer.2020.108077

[38] D. Rubenstein , W. Yin , M. Frame , in Biofluid Mechanics, 3rd ed. (Ed: K. Birtcher ), Academic Press, USA 2022, Ch. 11, pp. P423–442.

[39] J. Sun , Z. Wang , C. Yang , Crit. Rev. Anal. Chem. 2022, 1, 10.1080/10408347.2022.2139589. 36325979

[40] K. Johnson , M. Gregus , A. Ivanov , J. Proteome Res. 2022, 21, 2453. 36112031 10.1021/acs.jproteome.2c00337 PMC10118849

[41] L. Niu , P. Geyer , R. Gupta , A. Santos , F. Meier , S. Doll , N. Wewer Albrechtsen , S. Klein , C. Ortiz , F. Uschner , R. Schierwagen , J. Trebicka , M. Mann , Mol Syst Biol 2022, 18, e10947. 35579278 10.15252/msb.202210947 PMC9112488

[42] B. Loeys , H. Dietz , A. Braverman , B. Callewaert , J. De Backer , R. Devereux , Y. Hilhorst‐Hofstee , G. Jondeau , L. Faivre , D. Milewicz , R. Pyeritz , P. Sponseller , P. Wordsworth , A. De Paepe , J Med Genet 2010, 47, 476. 20591885 10.1136/jmg.2009.072785

[43] D. Guo , E. Hostetler , Y. Fan , R. Kulmacz , D. Zhang , D. Nickerson , S. Leal , S. Lemaire , E. Regalado , D. Milewicz , J Am Coll Cardiol 2017, 70, 2728. 29169482 10.1016/j.jacc.2017.09.1094 PMC6047350

[44] L. Tan , Z. Li , C. Zhou , Y. Cao , L. Zhang , X. Li , K. Cianflone , Y. Wang , D. Wang , Hum. Mol. Genet. 2017, 26, 4814. 28973303 10.1093/hmg/ddx360

[45] C. Villamizar , E. Regalado , V. Fadulu , S. Hasham , P. Gupta , M. Willing , S. Kuang , D. Guo , A. Muilenburg , R. Yee , Y. Fan , J. Towbin , J. Coselli , S. Lemaire , D. Milewicz , Eur J Med Genet 2010, 53, 80. 19941982 10.1016/j.ejmg.2009.11.001 PMC4354948

[46] K. Akutsu , H. Morisaki , S. Takeshita , H. Ogino , M. Higashi , T. Okajima , T. Yoshimuta , Y. Tsutsumi , H. Nonogi , T. Morisaki , Am. J. Cardiol. 2009, 103, 1146. 19361604 10.1016/j.amjcard.2008.12.037

[47] R. Suwal , S. Khadka , P. Joshi , Clin Ophthalmol 2020, 14, 2463. 32904572 10.2147/OPTH.S269364 PMC7457576

[48] M. Heur , B. Costin , S. Crowe , R. Grimm , R. Moran , L. Svensson , E. Traboulsi , Am. J. Ophthalmol. 2008, 145, 997. 18378212 10.1016/j.ajo.2008.01.028

[49] R. Zhang , K. Tiedemann , M. Muthu , N. Dinesh , S. Komarova , B. Ramkhelawon , D. Reinhardt , Cell. Mol. Life Sci. 2022, 79, 314. 35606547 10.1007/s00018-022-04337-8 PMC11072253

[50] F. D'Amico , E. Doldo , C. Pisano , M. Scioli , F. Centofanti , G. Proietti , M. Falconi , F. Sangiuolo , A. Ferlosio , G. Ruvolo , A. Orlandi , Int. J. Mol. Sci. 2020, 21, 6886. 32961817 10.3390/ijms21186886 PMC7555983

[51] S. Hibender , S. Li , A. Postma , M. Hoogeland , D. Klaver , R. Pouw , H. Niessen , A. Driessen , D. Koolbergen , C. De Vries , M. Baars , A. Houweling , P. Krijnen , V. De Waard , Vasc Biol 2022, 4, 40. 36279189 10.1530/VB-22-0016 PMC9782404

[52] A. Pedroza , Y. Tashima , R. Shad , P. Cheng , R. Wirka , S. Churovich , K. Nakamura , N. Yokoyama , J. Cui , C. Iosef , W. Hiesinger , T. Quertermous , M. Fischbein , Arterioscler Thromb Vasc Biol 2020, 40, 2195. 32698686 10.1161/ATVBAHA.120.314670 PMC7484233

[53] M. Jensen , A. Bonna , S. Frederiksen , S. Hamaia , P. Højrup , R. Farndale , H. Karring , Biochim Biophys Acta Proteins Proteom 2022, 1870, 140771. 35306228 10.1016/j.bbapap.2022.140771

