# Supplementary material for: Uncovering the Hidden World of Aqueous Humor Proteins for Discovery of Biomarkers for Marfan Syndrome
Source: Adv Sci (Weinh). 2023 Dec 13;11(6):2303161. doi: 10.1002/advs.202303161 (PMC10853735; doi:10.1002/advs.202303161)

ADVANCED SCIENCE
Open Access

Supporting Information

for *Adv. Sci.*, DOI 10.1002/advs.202303161

Uncovering the Hidden World of Aqueous Humor Proteins for Discovery of Biomarkers for Marfan Syndrome

Yumeng Shi, Jiahui Chen, Lei Cai, Xueling Zhang, Zexu Chen, Jin Yang, Yongxiang Jiang* and Yi Lu**

Figure S1 Quality Control (QC) Validation of Mass Spectrometer (MS) Data of AH

A-C) Peptides' length, numbers, and proteins' molecular weight distribution in AH. D) Box plot showing the distribution of Log10-transformed intensity values across AH samples. Samples with similar mean values are represented by a horizontal line, indicating good sample quality. E) Density distribution curve showing the peak value representing the maximum number of proteins at a given intensity level. The x-axis represents the intensity values of proteins after Log10 transformation, and the y-axis represents the probability density for all AH samples. F) Stacked bar plot showing the percentage of proteins expressed in different intensity ranges across all samples. G) OPLS-DA plot for 2,300 proteins in AH distinguishing MFS from CC. H) Bar graph showing the comprehensive functional annotation of the identified proteins in AH, including GO, protein domain, KEGG pathway, COG/KOG functional classification, subcellular localization, Reactome, WikiPathways, HallMark, and transcription factor (TF). AH, aqueous humor; CC, cataract controls; COG/KOG, Clusters of Orthologous Groups of proteins/euKaryotic Ortholog Groups; GO, Gene Ontology; KEGG, Kyoto Encyclopedia of Genes and Genomes; MFS, Marfan syndrome; OPLS-DA, Orthogonal partial least squares-discriminant analysis.

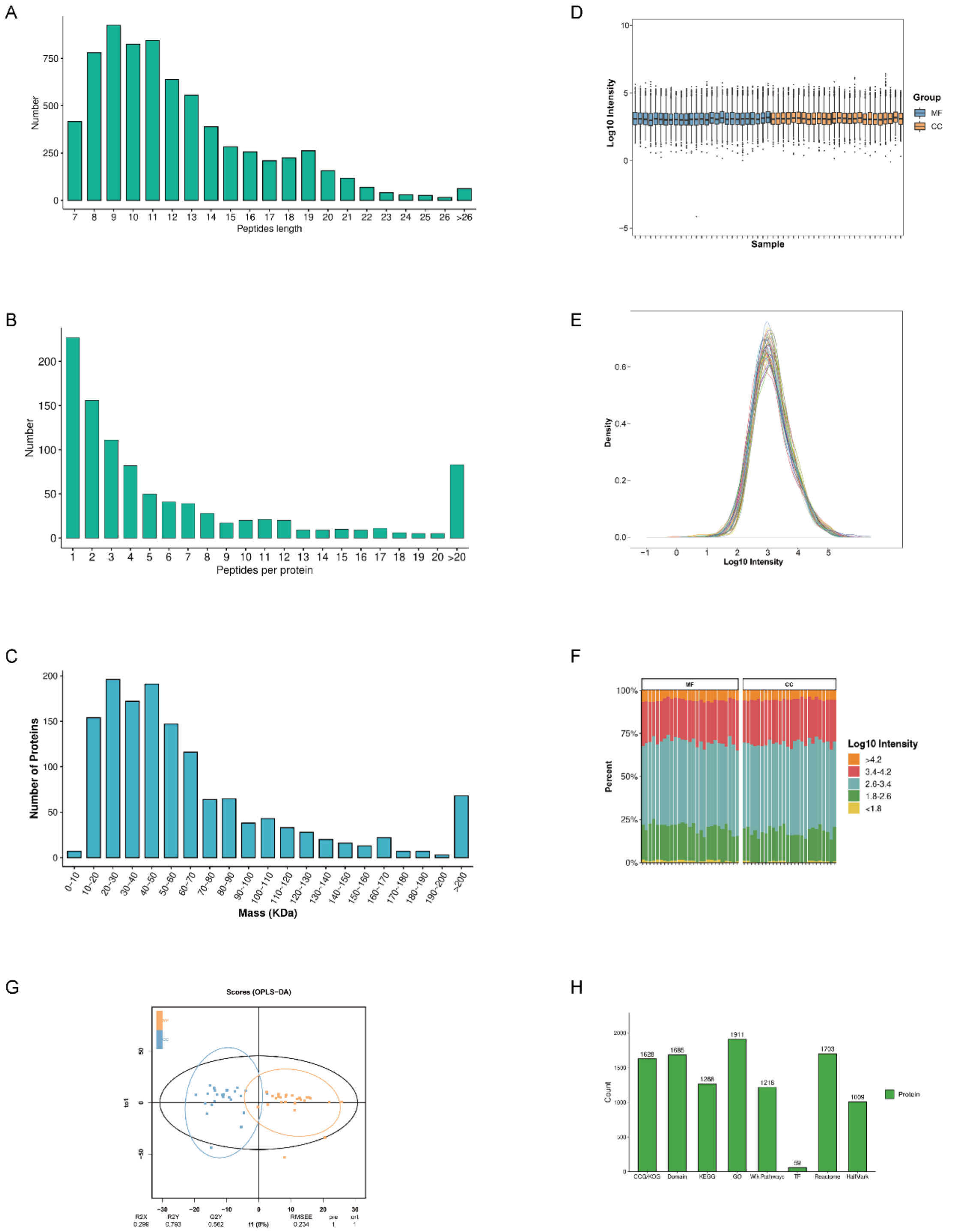

Figure S2 QC Validation of MS Data of Lens Capsule

A-C) Peptides' length, numbers, and proteins' molecular weight distribution in lens capsule. D) Box plot showing the distribution of Log10-transformed intensity values across lens capsule samples. E) Density distribution curve showing the peak value representing the maximum number of proteins at a given intensity level. The x-axis represents the intensity values of proteins after Log10 transformation, and the y-axis represents the probability density for all lens capsule samples. F) Stacked bar plot showing the percentage of proteins expressed in different intensity

ranges across all samples. G) Pie chart showing the distribution of protein coverage in lens capsule. H) Bar graph showing the comprehensive functional annotation of the identified proteins in lens capsule, including GO, Protein domain, KEGG pathway, COG/KOG functional classification, subcellular localization, Reactome, WikiPathways, HallMark, and transcription factor (TF). COG/KOG, Clusters of Orthologous Groups of proteins/euKaryotic Ortholog Groups; GO, Gene Ontology; KEGG, Kyoto Encyclopedia of Genes and Genomes.

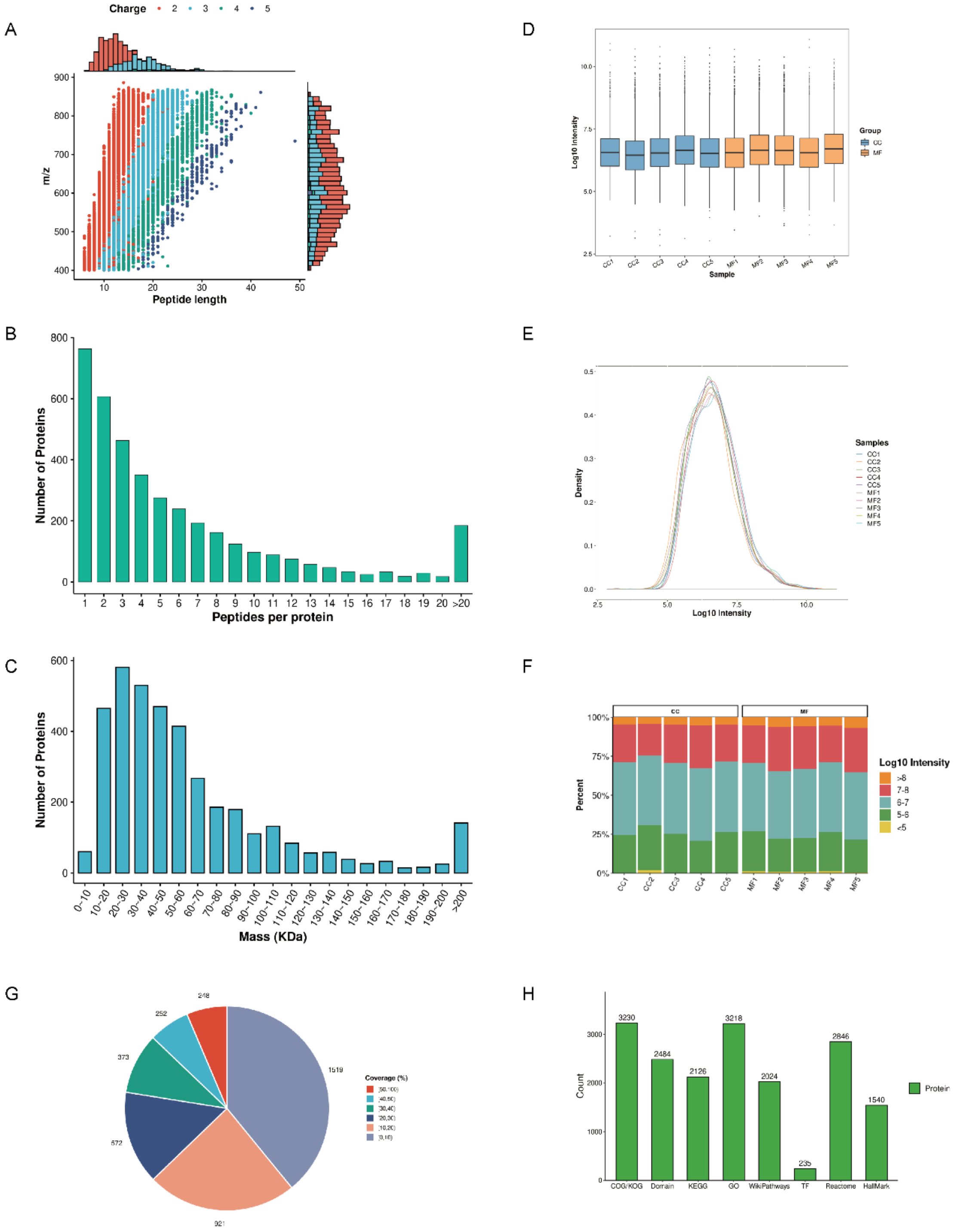

Supplement: Supplementary file 1 — Supporting Information [file ADVS-11-2303161-s003.pdf]
